# Genome-Wide Identification of the *WD40* Gene Family in Walnut (*Juglans regia* L.) and Its Expression Profile in Different Colored Varieties

**DOI:** 10.3390/ijms26031071

**Published:** 2025-01-26

**Authors:** Ruimin Xi, Jiayu Ma, Xinyi Qiao, Xinhao Wang, Hang Ye, Huijuan Zhou, Ming Yue, Peng Zhao

**Affiliations:** 1 Key Laboratory of Resource Biology and Biotechnology in Western China, Ministry of Education, College of Life Sciences, Northwest University, Xi’an 710069, China; xiruimin0328@163.com (R.X.); majiayu@stumail.nwu.edu.cn (J.M.); qiaoxinyi@stumail.nwu.edu.cn (X.Q.); xinhaowang@stumail.nwu.edu.cn (X.W.); sxsdyehang@163.com (H.Y.); 2 Xi’an Botanical Garden of Shaanxi Province, Institute of Botany of Shaanxi Province, Xi’an, Shaanxi Academy of Science, Xi’an 710061, China; zhouhuijuan@xab.ac.cn (H.Z.); yueming@nwu.edu.cn (M.Y.)

**Keywords:** *JrWD40*, anthocyanin, color regulation, red walnut

## Abstract

The walnut (*Juglans regia*) is a woody oilseed crop with high economic and food value as its kernels are edible and its hulls can be widely used in oil extraction and plugging, chemical raw materials, and water purification. Currently, red walnut varieties have emerged, attracting consumer interest due to their high nutritional values as they are rich in anthocyanins. *WD40* is a widespread superfamily in eukaryotes that play roles in plant color regulation and resistance to stresses. In order to screen for *JrWD40* associated with walnut color, we identified 265 *JrWD40s* in walnuts by genome-wide identification, which were unevenly distributed on 16 chromosomes. According to the phylogenetic tree, all *JrWD40s* were classified into six clades. WGD (Whole genome duplication) is the main reason for the expansion of the *JrWD40* gene family. *JrWD40s* were relatively conserved during evolution, but their gene structures were highly varied; lower sequence similarity may be the main reason for the functional diversity of *JrWD40s*. Some *JrWD40s* were highly expressed only in red or green walnuts. In addition, we screened 16 unique *JrWD40s* to walnuts based on collinearity analysis. By qRT-PCR, we found that *JrWD40-133*, *JrWD40-150*, *JrWD40-155*, and *JrWD40-206* may regulate anthocyanin synthesis through positive regulation, whereas *JrWD40-65*, *JrWD40-172*, *JrWD40-191*, *JrWD40-224*, and *JrWD40-254* may inhibit anthocyanin synthesis, suggesting that these JrWD40s are key genes affecting walnut color variation.

## 1. Introduction

Anthocyanins are secondary metabolites of flavonoids and different types of anthocyanins and their accumulation in the vesicles determine the different colors of flowers [1]. Flavonoid compounds provide color and flavor to fruits, flowers, and seeds, thereby attracting insects, birds, or mammals and facilitating pollen and seed dispersal [2]. In addition, it also protects plants from abiotic stresses such as strong light [3], UV light [4], and oxidative stress [5] by activating different flavonoid pathway genes. Flavonoids are mainly classified as chalcones, flavanones, isoflavones, flavonols, anthocyanins, and proanthocyanidins [6]. Anthocyanins are the main regulators of plant color, with cyanidin and peonidin causing purplish red in plants [7]. The anthocyanin synthesis pathway has been intensively studied in many plants, such as *Arabidopsis thaliana* [8,9], *Vitis vinifera* [10], and *Malus Domestica* [11]. Abiotic stresses such as temperature and light can regulate anthocyanin biosynthesis in fruit trees [10,11]. Flavonoid biosynthetic pathway is catalyzed by structural genes including *PAL*, *C4H*, *4CL*, *CHS*, *CHI*, *F3H*, *F3′H*, *F3′5′H*, *DFR*, *ANS*, and *UFGT*, which lead to the synthesis of anthocyanins [8]. Anthocyanins are mainly located in plant vesicles and are usually found in the form of geranins, cornflowerins, delphinids, paeoniflorins, petunids, and mallowins [12]. Furthermore, overexpression of *MYB* leads to increased *bHLH* and *TTG1* (*WD40*) expression, resulting in increased anthocyanin content [13]. The pigment overaccumulation resulting from the overexpression of *MYB* is dependent on *bHLH/TTG1*, and gene activation requires all members of the MBW (MYB-bHLH-WD40) complex, which consisted of *MYB*, *bHLH*, and *WD40* [14,15]. It is shown that MBW transcriptional complex influences phycocyanin synthesis through interactions [13,14,15]. In eggplants, anthocyanin biosynthesis is activated by the MYB-AN1-WD40 (MBW) complex or by *AN1* activation downstream of regulation through the MYB-JAF13-WD40 (MBW) complex, thereby affecting fruit color [16].

Among them, the WD protein family is widely distributed in eukaryotes [17]. The GTS1 folds into a β-propeller with seven pseudo-symmetrically arranged blades around the central axis and is first isolated in the β-subunit of G protein and CDC4 protein [18]. It possesses 4–16 tandemly conserved WD motifs, with each motif containing 40–60 amino acids with glycine-histidine (Gly-His, GH) at the N-terminus and tryptophan-aspartic acid (Trp-Asp, WD) at the C terminus [19]. The WD40 proteins are involved in a wide range of cellular functions, such as signal transduction, vesicular trafficking, cytoskeletal assembly, cell cycle control, apoptosis, chromatin dynamics, and transcription regulation [18]. The stable structure enables the WD40 protein to play an important role in plant development [20] and stress tolerance [21,22,23] and to regulate anthocyanin and proanthocyanidin biosynthesis [24]. Mutations in the *HOS15* gene (a type of WD40) lead to cold hypersensitive responses in plants [21,22]. Plants with deletion of the *CYP71* (a type of WD40) gene exhibit growth defects such as reduced meristematic tissues, lateral organ development, and reduced root elongation [25]. *WD40* activates the production of anthocyanins and proanthocyanidins mainly through the formation of MBW transcriptional aggregates with *MYB* and *bHLH* [24]. To date, the function of *WD40* has been extensively explored in a variety of plants, including *Arabidopsis thaliana*, tobacco (*Nicotiana tabacum*) [26], cotton (*Gossypium hirsutum*) [27], rice (*Oryza sativa*) [28], peach (*Prunus persica*) [29], mango (*Mangifera indica*) [29], and cucumber (*Cucumis sativus*) [30]. *MiTTG1* could respond to abiotic stresses, thus promoting root growth and development in mangoes [29]. *GIGANTUS1* and *XIW1* were associated with seed germination and regulated by ABA (Abscisic acid) response, respectively [30,31]. Overexpression of *SaTTG1* strains resulted in earlier flowering and a reduced number of rosette leaves. In addition, the seed size of the overexpression lines exceeded that of the wild type, indicating that overexpression of *SaTTG1* in *A. thaliana* could promote early flowering and seed enlargement [32]. Knockdown of the *NtTGG1* gene resulted in less leaf chlorosis in *NtTGG1*-silenced plants due to drought treatment compared to the wild type, suggested that silencing of *NtTGG1* enhances drought tolerance in tobacco [26]. *BvWD40-82* maintains ionic balance in cells by enhancing osmotic pressure levels and antioxidant enzyme activities, thereby improving salt tolerance in sugar beet (*Beta vulgaris*) and promoting seedling growth [33]. *FcWD40-97* (*FcTTG1*) interacts with *FcMYB114*, *FcMYB123*, and *FcbHLH42* to form the MBW transcriptional complex that promotes anthocyanin accumulation in figs (*Ficus carica*) [34]. *SmWD40-56* may play roles in formation of eggplant (*Solanum melongena*) fruit color by regulating anthocyanins through interactions with structural genes related to anthocyanin biosynthesis, affecting chalcone isomerase activity, oxidoreductase activity, and flavanone 3-hydroxylase activity [16].

The walnut (*Juglans regia* L.) is an important ecological and economic tree species. Walnuts have become increasingly popular among consumers in recent years. In addition to direct consumption, walnuts are also processed into walnut oil, protein powder, and walnut leisure snacks. Walnut hulls are widely used in oil mining plugging, chemical materials, water purification, and so on [35,36]. Walnut hulls boiled in water can treat pharyngitis, stomach ulcers, and other diseases [36]. Walnut trunks are also used as furniture, timber, and so on. Walnuts are widely planted, not only to increase the income of fruit farmers but also to prevent wind and sand [35]. Notably, color has gradually become an important external quality of walnuts, whose fruit, when it has an attractive appearance, can capture more consumer’s attention and thus obtain greater economic value. The anthocyanins of walnuts are found mainly in the leaves and fruits. In walnut leaves, there are 26 anthocyanins and 4 proanthocyanidins. Among them, 3-O-galactoside anthocyanins, 3-O-galactoside anthocyanins, 3-O-arabinoside anthocyanins, and 3-O-glucoside anthocyanins, as well as proanthocyanidins C1, B1, and B3 are the main anthocyanins and proanthocyanidins affecting the color of walnut leaves, respectively [37]. The walnut fruit is mainly composed of anthocyanins, with a low content of proanthocyanidins [38]. The color of the walnut fruit is mainly determined by the accumulation degree of chlorophyll, anthocyanin, carotene, and betaine. The ‘Zijing’ (ZJ) walnut variety found in Beijing, China, has purple branches, flowers, husks, and seed coat [39]. The ‘Lvling’ walnut (LL) is a common walnut with green leaves and hulls, and a light yellowish or brown seed coat [40]. The LL walnuts with large fruits and kernels and rich in oils and proteins are widely grown in China and are popular with consumers [41]. According to the current study, the MBW complex is the key factor in the process of anthocyanin accumulation in plants [42], and the walnut *MYB* and *bHLH* gene families, as well as the key *JrMYBs* and *JrbHLHs* affecting anthocyanin accumulation, have been revealed [43,44], whereas no studies have been conducted to identify *JrWD40* in a genome-wide study. It has been shown that some of the *JrWD40* genes respond to different temperatures [45]. However, it has not been investigated whether *WD40* affects walnut color and which *JrWD40* is the key gene that affects walnut color.

Hence, we conducted a genome-wide identification of the *JrWD40* members, further analyzed the chromosomal location and collinearity, phylogenetic, structural and *cis*-acting elements, protein–protein interaction, and microRNA target prediction. In particular, RNA-seq data were used to analyze *JrWD40s* expression in different colored cultivar leaves and peels. Combined with qRT-PCR experiments, the 16 unique *JrWD40* genes were analyzed for their expression patterns in ZJ and LL leaves and peels. It lays the foundation for the screening of key *WD40* affecting anthocyanin accumulation and provides scientific theoretical support for the breeding of walnut varieties with better coloration.

## 2. Results

### 2.1. Phylogenetic Analysis of All JrWD40s

We identified a total of 265 *WD40* gene family members in walnuts. To enable follow-up studies, we renamed *JrWD40s* in relation to the order in which these genes are located on the chromosomes. All identified *JrWD40* member names, gene IDs, and protein sequences were displayed in Table S1.

**Figure 1:**
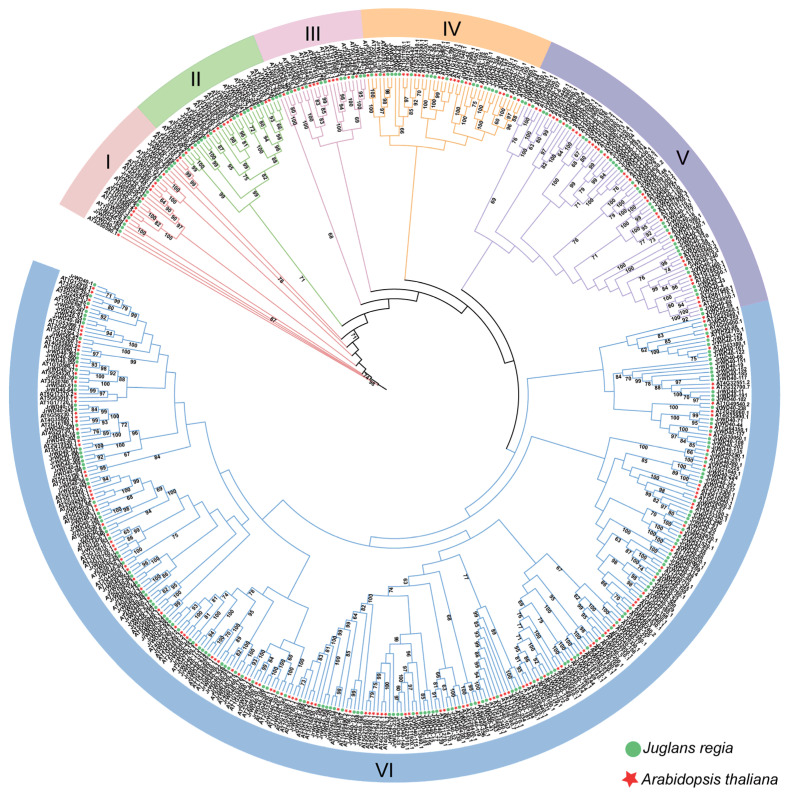
Phylogenetic relationships of 265 *JrWD40s* and 230 *AtWD40s*.

Subsequently, we used 265 identified *JrWD40s* and 230 *AtWD40s* constructed phylogenetic trees by the maximum likelihood method (Figure 1). All *WD40s* were grouped into six clades (Clade I–VI) in accordance with phylogenetic analysis. All six clades contained both *Arabidopsis* and *J. regia WD40* members and are relatively evenly distributed, suggesting that they are closely related to each other and relatively conserved in the course of evolution. Therein, the largest clade (Clade VI), contained 165 *JrWD40s* and 138 *AtWD40s*. Next is Clade V, which contained 36 *JrWD40s* and 37 *AtWD40s.* Clade IV contained 21 *JrWD40s* and 20 *AtWD40s*. Clade II contained 17 *JrWD40s* and 12 *AtWD40s*. Clade I contained 14 *JrWD40s* and 12 *AtWD40s*. The smallest clade is Clade III, which contained only 12 *JrWD40s* and 11 *AtWD40s*. Interestingly, we also found longer evolutionary branches between individual gene pairs (*At3G50590.1*, *JrWD40-94*, and *JrWD40-190*; *JrWD40-233*, *At5G52820.1*, and *JrWD40-227*), suggesting that these genes may have evolved with greater mutations.

### 2.2. Characterization of the JrWD40s

**Figure 2:**
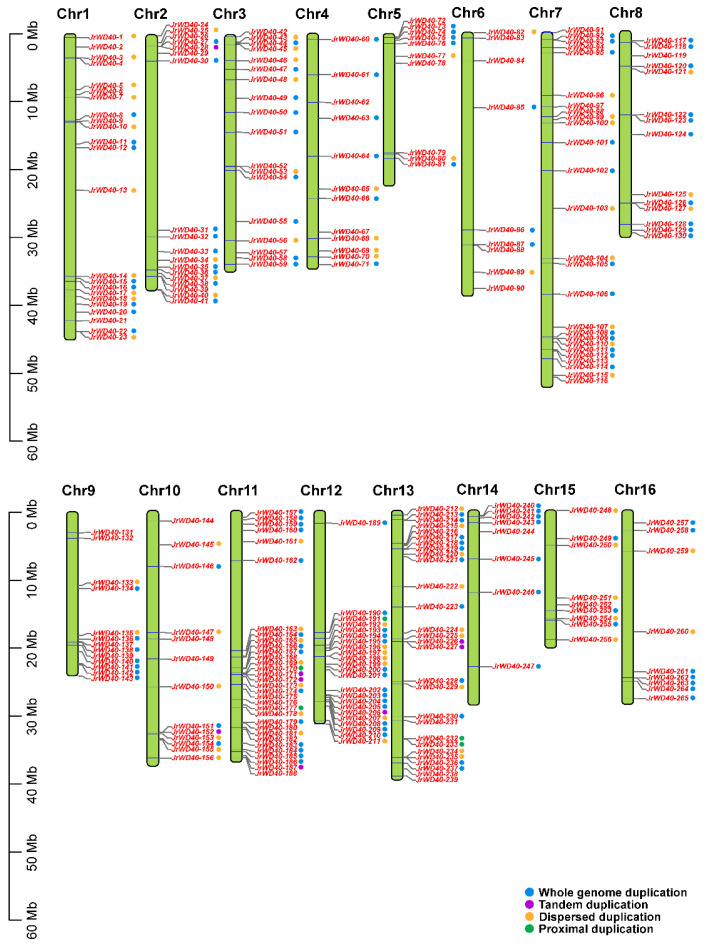
Chromosomal positional distribution and gene duplication pattern of *JrWD40s.* The blue, purple, yellow, and green circles represent WGD, TD, DSD, and PD, respectively.

Chromosomal localization analysis displayed that all *JrWD40s* were distributed on 16 chromosomes, but the distribution was not uniform (Figure 2). Chromosome 11 has the largest number of *JrWD40s* with 32 and a gene density of 12.08%. This is followed by chromosome 13 and chromosome 7 with 28 *JrWD40s* and 26 *JrWD40s*, respectively, the former with a gene density of 10.57% and the latter with a gene density of 9.81%. Some chromosomes contained relatively small numbers of *JrWD40s*, such as chromosome 15 and chromosome 16, with only 9 *JrWD40s*. There are also regions with relatively high densities of chromosomal *JrWD40s*, such as the ends of the chromosomes 1, 2, 7, 9, 11, 12, and 13.

The structural domains of *JrWD40s* are relatively conserved (Figure S2). All identified *JrWD40s* contained the WD40 domain and WD40 superfamily domain, but *WD40* is different in sequence length, number of repetitions, and so on. There are also some *JrWD40s* that contained other domains, such as F-box, Ubox, Lish, RAD, and WDAD. The results of conserved motifs showed that *JrWD40s* were detected in 10 conserved motifs (Figure S3). The number of amino acids per motif varied from 15 to 50 (Figure S4). Almost all *JrWD40s* contained motif 2 (except JrWD40-13, 38, 62, 84, 89, 98, 122, 129, 155, 158, 167, 171, 182, 211, and 226). It is worth mentioning that individual *JrWD40s* contained only one motif. As an example, *JrWD40-38* contained only motif 6, *JrWD40-62* contained only motif 1, and *JrWD40-89* and *JrWD40-155* contained only motif 3. *JrWD40s* vary widely in gene structure, with exon numbers ranging from 1 (JrWD40 to 1, 7, *27*, *28*, *30*, *65*, *74*, *85*, *95*, *109*, *134*, *150*, *168*, *183*, *189*, *204*, *215*, *223*, *224*, and *228*) to 31 (*JrWD40-86* and *JrWD40-253*) and individual genes contained long introns (Figure S5). These results may be due to the low protein sequence similarity of *WD40* gene family members. However, this may also be the main reason for the functional diversity of the *WD40* gene family.

The number of amino acids in 265 JrWD40s ranged from 150 aa (JrWD40-155 and JrWD40-226) to 3613 aa (JrWD40-163), with an average of about 676 aa (Table S3). Molecular weight ranged from 16,576.9 Da (JrWD40-226) to 402,714.14 Da (JrWD40-163), with an average of 78,432.17 Da. There are 189 JrWD40s that were acidic proteins and 76 JrWD40s that are basic proteins. The 91 JrWD40s with instability indices less than 40 and 174 JrWD40s with greater than 40 indicate that only about 34.34% of JrWD40s are stable proteins and the vast majority of JrWD40s are unstable. In addition, only nine JrWD40s had a grand average of hydropathicity (GRAVY) greater than zero, indicating that the vast majority of JrWD40s are hydrophilic. *JrWD40s* are widely distributed in subcellular structures such as peroxisome, plasma membrane, chloroplast, endoplasmic reticulum, cytoplasm, cytoskeleton, exocytosis, mitochondria, and vesicles, but the majority of *JrWD40s* are still localized in nucleus.

### 2.3. Synteny Analysis of JrWD40s

**Figure 3:**
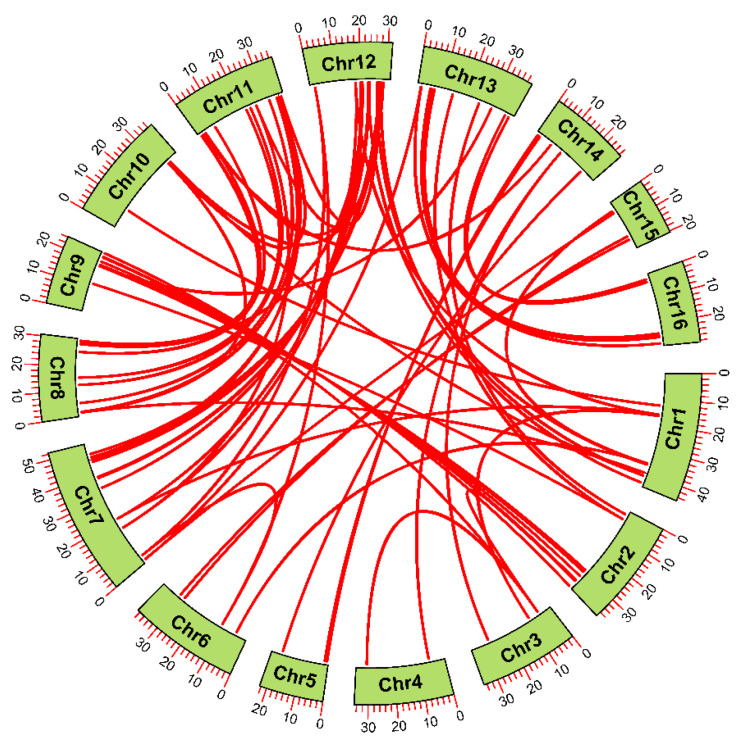
Collinearity analysis of *JrWD40s*. Red lines connect homologous genes.

Collinearity analysis showed that *JrWD40s* had 75 paralogous gene pairs (Figure 3). These results suggest that gene duplication events may have occurred in some *JrWD40s.* We then predicted the gene duplication patterns of all identified *JrWD40s* (Figure 2, Table S4), according to which 128 *JrWD40s* had WGD events, 77 *JrWD40s* had DSD (Dispersed duplication), 8 *JrWD40s* had TD (Tandem duplication), and 5 *JrWD40s* had PD (Proximal duplication, Figure S6). It was shown that WGD was the major duplication pattern of *JrWD40s*. However, there are still some *JrWD40s* that do not undergo duplication events, possibly due to the fact that after gene duplication in some genes, one of the members of a gene pair is genetically eliminated from the genome, thus returning the gene to a singleton state. To further investigate the selection pressure on *JrWD40* gene pairs, we measured *Ka/Ks* ratios of these 75 paralogous *JrWD40* gene pairs, all of which were significantly less than one, suggesting that all of these *JrWD40s* pairs underwent selection by purification during the evolutionary process (Table S5).

**Figure 4:**
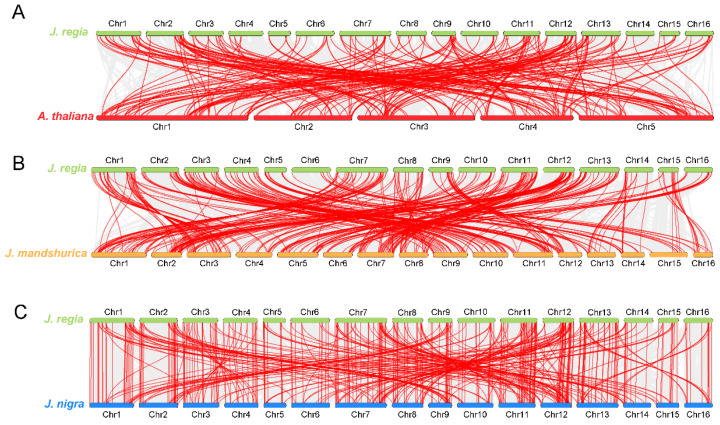
Collinearity analysis of *WD40s*. (**A**) Collinearity analysis between *JrWD40s* and *Arabidopsis thaliana*; (**B**) Collinearity analysis between *JrWD40s* and *Juglans mandshurica*; (**C**) Collinearity analysis between *JrWD40s* and *Juglans nigra*.

To explore possible evolutionary processes of *WD40s*, we enforced collinearity analysis between walnuts, *Arabidopsis*, and two walnut relatives *J. mandshurica* and *J. nigra* (Figure 4). *JrWD40s* had 219 homologous *WD40* gene pairs with *Arabidopsis* (Figure 4A, Table S6), 384 homologous *WD40* gene pairs with *J. mandshurica* (Figure 4B, Table S7), and 423 homologous *WD40* gene pairs with *J. nigra* (Figure 4C, Table S8). The two walnut relatives have more homologous gene pairs compared to *Arabidopsis* with *JrWD40s*, suggesting closer relationships among the three *Juglans* species. Notably, 16 *JrWD40s* (*JrWD40-65*, *JrWD40-133*, *JrWD40-150*, *JrWD40-155*, *JrWD40-169*, *JrWD40-171*, *JrWD40-172*, *JrWD40-177*, *JrWD40-187*, *JrW40-191*, *JrWD40-197*, *JrWD40-206*, *JrWD40-224*, *JrWD40-227*, *JrWD40-233*, and *JrWD40-254*) had no gene pairs with any of the selected species, indicating that these 16 *JrWD40s* may be walnut-unique genes.

### 2.4. Cis-Acting Elements Analysis of JrWD40s

**Figure 5:**
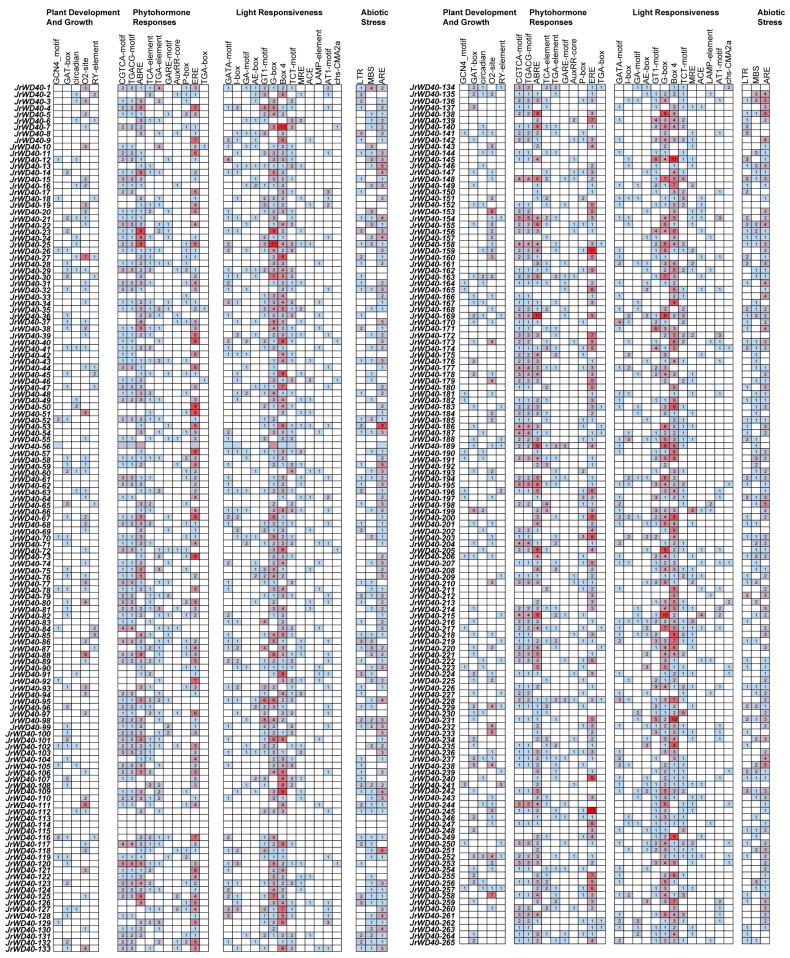
The *cis*-acting elements of *JrWD40s*. The different colors represent differences in the number of *cis*-acting elements. The redder the color, the greater the number of *cis*-acting elements, the opposite is true for blue.

To investigate the potential functions of *JrWD40s*, we predicted *cis*-acting elements for 265 *JrWD40s* promoter region. We focused on four categories of *cis*-acting elements related to response to plant development and growth, phytohormone, light, and abiotic stress (Figure 5). We found that *JrWD40s* contained a large number of elements that respond to estrogen (ERE), ABA (ABRE), light (G-box and box 4), and antioxidants (ARE). The *JrWD40-145* had 13 ERE elements and *JrWD40-7* and *JrWD40-159* had 11 ERE elements. The *JrWD40-169* had 11 ABREs and *JrWD40-23* and *JrWD40-189* had 9 ABRE elements, respectively. The *JrWD40-25* had 11 G-box elements and *JrWD40-7* and *JrWD40-145* had 11 box 4 elements. The *JrWD40-253* had 9 ARE elements. Almost all *JrWD40s* contained elements that respond to phytohormone, light, and abiotic stress, implying that *JrWD40s* may play pivotal parts in walnuts’ resistance to stresses.

### 2.5. Expression Profiles of JrWD40s in Different Colored Walnut Cultivars

**Figure 6:**
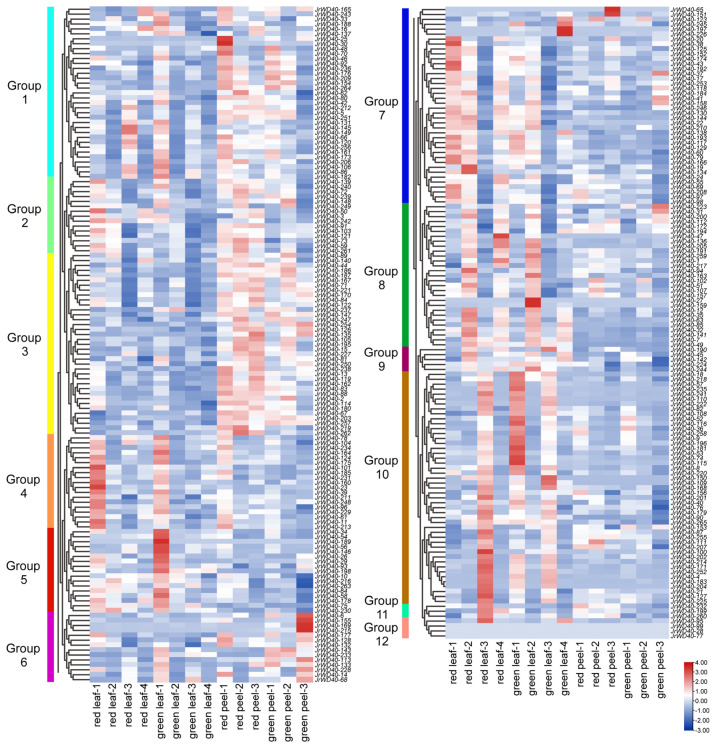
Expression profiles of *JrWD40s* in leaves and peels of different color walnut varieties. Leaf-1 represents expansion stage, leaf-2 represents new shoot growing stage, leaf-3 represents fruit swelling stage, and leaf-4 represents early period of fruit ripening. Peel-1, peel-2, and peel-3 represent those collected 30, 60, and 90 days after flowering, respectively. Colored bars indicated groups clustered by their expression patterns.

To examine the role of *JrWD40s* in regulating the different colors of walnuts, we analyzed the expression of all identified 265 *JrWD40s* in the leaves and peels of red walnuts (RW-1) and green walnuts (zhonglin-1, Figure 6; Table S9). Based on their different expression profiles, these *JrWD40s* were clustered into 12 subgroups. Three of these *JrWD40s* (group 12; *JrWD40-28*, *JrWD40-77*, and *JrWD40-99*) were not expressed in red and green walnuts leaves and peels at all periods, and these three *JrWD40s* may function in other tissues or in other varieties. The *JrWD40s* in group 7 were highly expressed in the early leaves of red walnuts, and the *JrWD40s* in group 5 were high expressed in the early leaves of green walnuts. Most of the *JrWD40s* in groups 1, 2, and 3 were highly expressed in the peels of red walnuts. This suggests that these genes may be participating in regulatory processes that affect the different colors of walnuts. The expression of *JrWD40s* was higher in the peels than in leaves of both walnut varieties in group 3. The expression of *JrWD40s* was greater in leaves than in peels in groups 9 and 10. These *JrWD40s* may be tissue specific. In addition, there were some genes that may be related to the growth and development of walnuts. For example, *JrWD40s* in group 4 were strongly expressed in the first stage of the leaves of both walnut varieties, suggesting that these *JrWD40s* may play roles in young walnut leaves.

### 2.6. Expression Profiles, Protein Interactions, and miRNA Targeting Prediction of 16 Unique JrWD40s

**Figure 7:**
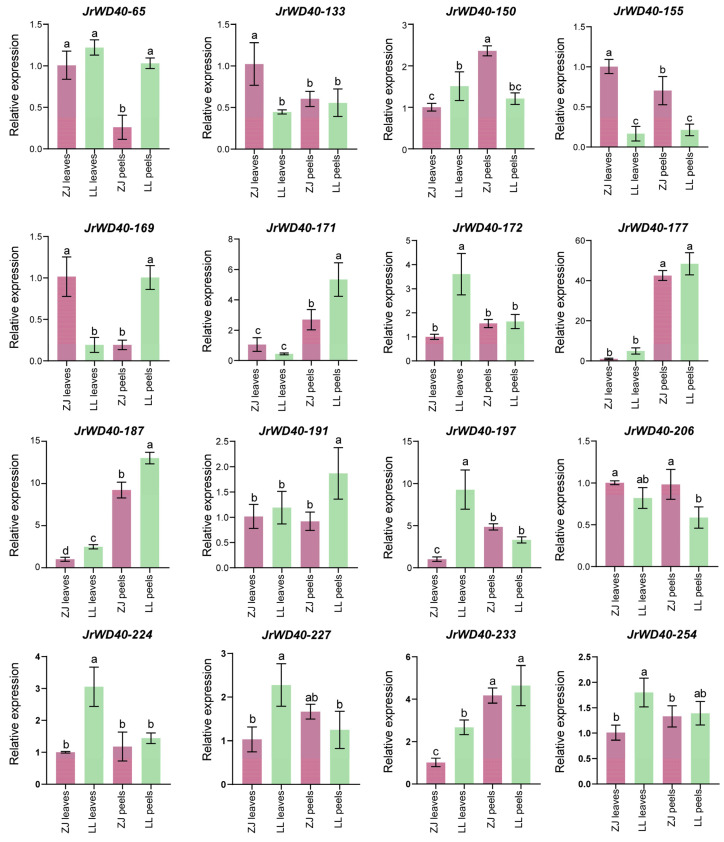
The qRT-PCR of 16 unique *JrWD40s* in ZJ and LL walnuts. The X-axis coordinates consecutively indicated the leaves of ZJ, the leaves of LL, the peels of ZJ, and the peels of LL. Different letters represent significant differences.

Based on the results of collinearity, 16 *JrWD40s* may be walnut-specific genes (Figure 4), so we analyzed the expression of 16 unique *JrWD40s* in leaves and peels of purplish walnut variety (ZJ) and the green walnut variety (LL) by qRT-PCR experiments (Figure 7). These 16 *JrWD40s* had different expression patterns in ZJ and LL walnuts. *JrWD40-133*, *JrWD40-150*, *JrWD40-155*, and *JrWD40-206* had a relatively high expression in the leaves and peels of ZJ. *JrWD40-65*, *JrWD40-172*, *JrWD40-191*, *JrWD40-224*, and *JrWD40-254* had a higher expression in the leaves and peels of LL than those of ZJ, demonstrating that these *JrWD40s* are candidate genes involved in the regulation of walnut color. *JrWD40-171*, *JrWD40-177*, *JrWD40-187*, and *JrWD40-233* had a higher expression in the peels of the walnuts, indicating that these four *JrWD40s* may be associated with peel development. *JrWD40-172* and *JrWD40-197* had similar expression patterns and both were highly expressed in the leaves of LL.

**Figure 8:**
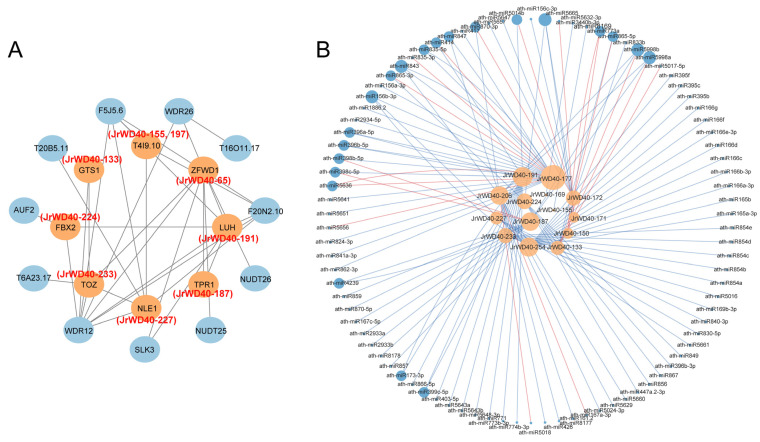
Network analysis of 16 unique *JrWD40s.* (**A**) Protein–protein interactions of the 16 unique JrWD40s; (**B**) miRNA targeting predictions of the 16 unique *JrWD40s*. Red line represent genes regulated by translation and blue lines represent genes regulated by cleavage.

Subsequently, we predicted the interaction relationships of these 16 unique JrWD40 proteins by homology analysis based on the interaction relationships of WD40 proteins in *A. thaliana* (Figure 8A). WDR12 and WDR26 had the most interactions with these 16 unique JrWD40s. WDR is also a subfamily of the *WD40* family, implying that the *WD40* subfamily interacts with each other to regulate walnut growth. A total of 2476 miRNAs targeted 259 *JrWD40s* (except *JrWD40-6*, *JrWD40-44*, *JrWD40-65*, *JrWD40-197*, *JrWD40-226*, and *JrWD40-255*; Table S10), of which a total of 116 miRNAs targeted 14 unique *JrWD40s* (except *JrWD40-65* and *JrWD40-197*; Figure 8B). Among these 116 miRNAs, 95 miRNAs regulated *JrWD40s* by cleavage and 21 regulated *JrWD40s* by translation. Thus, cleavage is the main mode of miRNA targeting *JrWD40s*.

## 3. Discussion

### 3.1. Characteristics of JrWD40s

The *WD40* family acts as a major player in the regulating of plant development [46], influencing fruit colors [16] and plant adversity stress [47]. It is a large gene family in eukaryotes. The walnut is a woody oilseed crop with substantial economic, food, and ecological value [48]. Unfortunately, studies on the walnut *WD40* family members have not been reported. During this study, we identified a total of 265 *JrWD40s* in walnuts. These *JrWD40s* were unevenly distributed across the 16 chromosomes of walnuts. The 265 *JrWD40s* and 230 *AtWD40s* were divided into six clades based on their phylogenetic relationships, and all six clades contained *WD40s* from both species, illustrating that *WD40s* were relatively conserved during evolution. However, *WD40s* are not conserved in sequence and structure. Although all of these 265 *JrWD40s* contained the WD40 domain and WD40 superfamily domain, there are still some *JrWD40s* that contain other domains, and their sequence lengths and number of repeats vary. Similar phenomena were found in tomatoes [49], potatoes [48], roses [50], etc. The number of exons in *JrWD40s* also varied from 1 to 31, and some *JrWD40s* contained very long introns. As non-coding regions, introns protect genes from mutations and maintain gene function [51]. The discrepancy of these sequences may be the main explanation for the functional heterogeneity of *WD40* family.

As predicted by physicochemical properties, 71.32% of *JrWD40s* were acidic proteins (189 out of 265), 65.66% of *JrWD40s* were unstable proteins (174 out of 265), and 96.6% of all *JrWD40s* were hydrophilic proteins (256 out of 265). Furthermore, *JrWD40s* were widely distributed in organelles and not just localized only in the nucleus. This is consistent with the phenomenon observed in peaches, where *WD40* members were localized in different locations, with *Prupe.6G21180.1* in the cell membrane, *Prupe.5G116300.1* in the nucleus and cell membrane, and *Prupe.8G212400.1* and *Prupe.1G053600.1* mainly in the nuclear periplasm and cell membrane [29].

Many *WD40s* have been shown to respond to abiotic stresses [23,52,53]. Almost all *JrWD40s* had *cis*-acting elements in response to growth, hormones, light, and abiotic stresses, especially those in response to estrogen (ERE), ABA (ABRE), light (G-box and box 4), and antioxidants (ARE), which imply that *JrWD40s* may play pivotal parts in walnuts’ resistance to stresses. *SmWD40s* contain a large number of light-responsive elements, indicating strong correlation between their gene expression and light [16]. A similar phenomenon was observed in walnuts.

### 3.2. Evolution of JrWD40s in Walnuts

*WD40* is a large gene family, but the number of members varies in different species. There were 230 *AtWD40s* in *A. thaliana*, 207 *SlWD40s* in *tomatoes* [49], 178 *StWD40s* in potatoes [48], 219 *PpWD40s* in peaches [52], and 177 *BvWD40s* in sugar beets [33]. We identified 265 *JrWD40s* in walnuts. Gene duplication had long been recognized as driver for organisms to evolve genes with new functions [54,55]. There were a large number of gene duplication events in *JrWD40s*, with WGD being the dominant mode of duplication in *JrWD40s*. There were 219 *WD40* homologous gene pairs in walnuts and *A. thaliana*, 384 homologous *WD40* gene pairs in *J. mandshurica*, and 423 homologous *WD40* gene pairs in *J. nigra*. Similar phenomenon was found in other gene families of walnut, such as *4CL* [48], *R2R3-MYB* [43], and *LEA* [56]. These results all suggest a closer collineaity between woody plants.

### 3.3. Potential JrWD40s Affecting the Color of Walnuts

Nowadays, some red walnuts have appeared in the market, and their special appearance and high nutritional value have attracted the attention of consumers. For example, ZJ walnuts have purplish-red colored leaves, peels, and all other organs, and “Hongren” walnuts have reddish seeds and seed coats. These cultivars increased the economic value of walnuts [44]. A large body of evidence demonstrates that the *WD40* genes may be broadly involved in the biosynthesis of plant anthocyanins, thereby affecting plant colors. Depending on the transcriptome results, *JrWD40s* showed different expression patterns in different colored walnut varieties, and some of them were highly expressed only in red or green walnuts, indicating that these *JrWD40s* may be involved in the synthesis of walnut anthocyanins, which affects the color of walnuts. According to the collinearity relationships, there were 16 unique *JrWD40s* in walnuts which had no homologues in the other three selected species. Among these 16 unique genes, *JrWD40s*, *JrWD40-133*, *JrWD40-150*, *JrWD40-155*, and *JrWD40-206* had a relatively high expression in the leaves and peels of ZJ. *JrWD40-65*, *JrWD40-172*, *JrWD40-191*, *JrWD40-224*, and *JrWD40-254* had a higher expression in the leaves and peels of LL than those of ZJ. These results denoted that these nine *JrWD40s* may be the key candidate genes that participated in walnut anthocyanin synthesis. In *R. simsii*, 17 potential candidate *RsWD40s* associated with anthocyanin synthesis were also targeted by qRT-PCR experiments [57]. In addition, these unique *JrWD40* proteins interact with WDR proteins to co-regulate walnut color. Similar results have been found in other species. The gene expression levels of *CHS*, *CHI*, *F3H*, and *DFR* were significantly reduced after silencing *SmWD40-56*, and it is speculated that *SmWD40-56* may interact with the above proteins to regulate anthocyanin synthesis-related genes, thus affecting the formation of eggplant fruit color [16]. Potato *PG0026477* interacts directly with structural genes such as *ANS*, *F3′5′H*, *DFR*, *F3H* and *FLS* to affect anthocyanin synthesis [47]. This study shows that *WD40* regulates the biosynthesis of walnut anthocyanins. It provides a valuable basis for the study of the molecular mechanism of walnut color change and also provides scientific theoretical support for the cultivation of walnut varieties with better coloring.

## 4. Materials and Methods

### 4.1. Genome-Wide Identification of JrWD40 Gene Members

To determine the *WD40* family members in *J. regia*, 237 *A. thaliana WD40* members were downloaded from the TAIR (The *Arabidopsis* Information Resource) [58] as query sequences and the BLASTP (E-value < 1 × 10^−5^) was performed for genome-wide identification in the walnut Chandler 2.0 reference genome. Following this, comparisons with the identified candidate *JrWD40s* were made by manual screening. The protein sequences of such potential *JrWD40s* were submitted to NCBI CDD (Conserved Domain Database) database [59]. We considered genes containing WD40 domain and WD40 superfamily domain as final *JrWD40* members.

### 4.2. Chromosomal Location and Collinearity Analysis of JrWD40s

Based on the genome annotation information, the identified *JrWD40* members were chromosomally localized using TBtools software (v1.0) [60] and renamed depending on their position along the chromosome (Table S1). Gene duplication events and collinearity predictions between *A. thaliana*, *J. mandshurica* [61], *J. nigra* [62], and *J. regia* were performed by MCascanX software (v1.0.0) [63] using default parameters.

### 4.3. Phylogenetic Analysis of JrWD40s

Maximum likelihood (ML) tree was created for WD40 proteins from *J.regia* and *Arabidopsis thaliana* by using the IQTREE software (v2.3.6) [64] (Bootstarp:1000; Best BIC score model: VT + F + G4). The results were then beautified with the online website iTOL (Interaction Tree of Life) [65]. Specific details can be found at [66].

### 4.4. Characteristic Information of JrWD40s

Protein sequences of identified JrWD40 members were submitted to the online websites ExPASy [67] and WoLF PSORT [68] for predicted physicochemical properties and subcellular locations, respectively. The CDD database was used for conserved domains analysis [59]. Conserved motifs in JrWD40s were identified by MEME Suite [69], uploading JrWD40s protein sequences and selecting found motif number = 10. The structures of *JrWD40s* were analyzed using GSDs [70]. An upstream 2000 bp of the gene transcriptional start site was identified as the promoter region, and *cis*-acting elements of all *WD40* genes were predicted by PlantCARE [71].

### 4.5. Interaction Network Map and miRNA Targeting of JrWD40s

Interaction networks were analyzed with the online website STRING [72] and by referring to the *Arabidopsis* protein database, with all identified JrWD40 sequences as query sequences. Then, they were visualized by Cytoscape software (v3.9.1) [73] with default parameters. All identified *WD40* members were submitted to the psRNA-Target website [74] for microRNA target prediction.

### 4.6. Gene Expressions and qRT-PCR Experiments of JrWD40s

Gene expression data for different color cultivars walnuts were obtained from NCBI (PRJNA688391) [44], where leaves and peels were taken from red and green walnuts (ZW-1 and Zhonglin-1). The low-quality reads were excluded from the raw reads, then using the HISAT2 [75], the reads to were mapped to the Chandler 2.0 genome and then the mapped reads were integrated using StringTie software (v2.2.1) with default parameters [76]. Gene expression levels were gauged regarding FPKM (fragments per kilobase of exon model per million mapped fragments) values using FeatureCounts software (v2.0.6) [77].

We collected leaves and peels of 8-year-old ZJ and LL walnuts at the Shaanxi Provincial Botanical Garden (Figure S1). Then, they were frozen quickly after collection using liquid nitrogen and stored at −80 °C. The total RNA of the samples was extracted by the Omega RNA Extraction Kit (Biel, Switzerland). Then, they were reverse transcripted using the Evo M-MLV RT Permix Kit (Cat No. AG11706, Accurate Biotechnology Co., ltd., Wuhan, China) to synthesize the first-strand cDNA as the template for the quantitative real-time polymerase chain reaction (qRT-PCR). The primers for *JrWD40s* were designed by Primer3 (Table S2) [78]. The qRT-PCR reactions were conducted in the CFX Real-Time PCR Detection System (Bio-Rad, Hercules, CA, USA) by using the SYBR^®^ Green Premix Pro Taq HS qPCR Kit (Cat No.AG11701, Accurate Biotechnology Co., ltd., Wuhan, China). The qRT-PCR reaction system of 10 μL SYBR Green Premix Pro Taq HS qPCR Kit, 0.5 μL of each forward and reverse primers, 2 μL cDNA, and ddH_2_O to 20 μL was used. The reaction was performed at 94 °C for 2 min of pre-denaturation, denaturation at 94 °C for 15 s, annealing at 58 °C for 15 s, extension at 72 °C for 30 s, and 40 cycles. Relative expression level of *JrWD40s* was calculated by the 2^−ΔΔCt^ method [66] and β-actin gene (*LOC108979429*) as an internal reference gene [56].

## 5. Conclusions

For this study, we identified 265 *JrWD40s* in a genome-wide analysis. These 265 *JrWD40s* were unevenly distributed on 16 chromosomes. Based on phylogenetic analysis, they were classified into six clades, which were relatively conserved during evolution. However, the sequence similarity of these *JrWD40s* was low, resulting in functional diversity. Candidate *JrWD40s* associated with walnut color were screened by transcriptome data of different colored walnut varieties. Pursuant to collinearity analysis, 16 *JrWD40s* that might be specific to walnuts were found, and their gene expressions in ZJ and LL walnuts were analyzed by qRT-PCR experiments. Among them, we found nine key *JrWD40s* affecting walnut color. *JrWD40-133*, *JrWD40-150*, *JrWD40-155*, and *JrWD40-206* may regulated anthocyanin synthesis through positive regulation, whereas *JrWD40-65*, *JrWD40-172*, *JrWD40-191*, *JrWD40-224*, and *JrWD40-254* may inhibit anthocyanin synthesis. These results provide a basis for further studies on the functions of *WD40s* in walnuts.

## Data Availability

The raw data were downloaded from the National Center for Biotechnology Information database under accession numbers (PRJNA688391).

## Supplementary Materials

The supporting information are available online at https://www.mdpi.com/article/10.3390/ijms26031071/s1.
