# Supplementary material for: Genome-Wide Identification of the WD40 Gene Family in Walnut (Juglans regia L.) and Its Expression Profile in Different Colored Varieties
Source: Int J Mol Sci. 2025 Jan 26;26(3):1071. doi: 10.3390/ijms26031071 (PMC11817448; doi:10.3390/ijms26031071)
Supplement: Supplementary file 1 [file ijms-26-01071-s001.zip › Figure captions.pdf]

Figure S1: Morphological characteristics of ZJ and LL walnuts. (A) Morphological diagram of LL fruit after ripening; (B) Morphological diagram of ZJ fruit after ripening; (C) Morphological diagram of ZJ leaves and fruits during development; (D) Morphological diagram of ZJ kernel;

Figure S2: Conserved domains visualization of 265 *JrWD40s*.

Figure S3: Conserved motifs visualization of 265 *JrWD40s*.

Figure S4: Conserved motifs and predicted structures of *JrWD40s*.

Figure S5: Gene structure of 265 *JrWD40s*.

Figure S6: *JrWD40s* gene duplication patterns number statistics by type.
