# Supplementary figures and images for: Genome-Wide Identification of the WD40 Gene Family in Walnut (Juglans regia L.) and Its Expression Profile in Different Colored Varieties
Source: Int J Mol Sci. 2025 Jan 26;26(3):1071. doi: 10.3390/ijms26031071 (PMC11817448; doi:10.3390/ijms26031071)

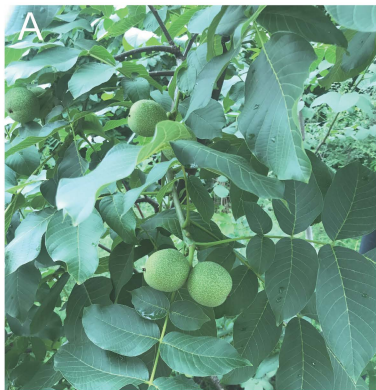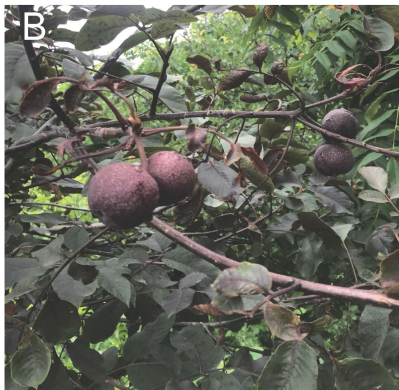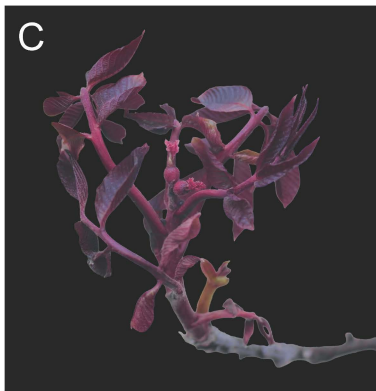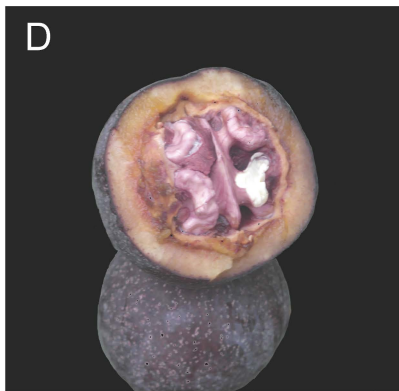

Supplement: Supplementary file 1 [file ijms-26-01071-s001.zip › Figure S1.pdf]

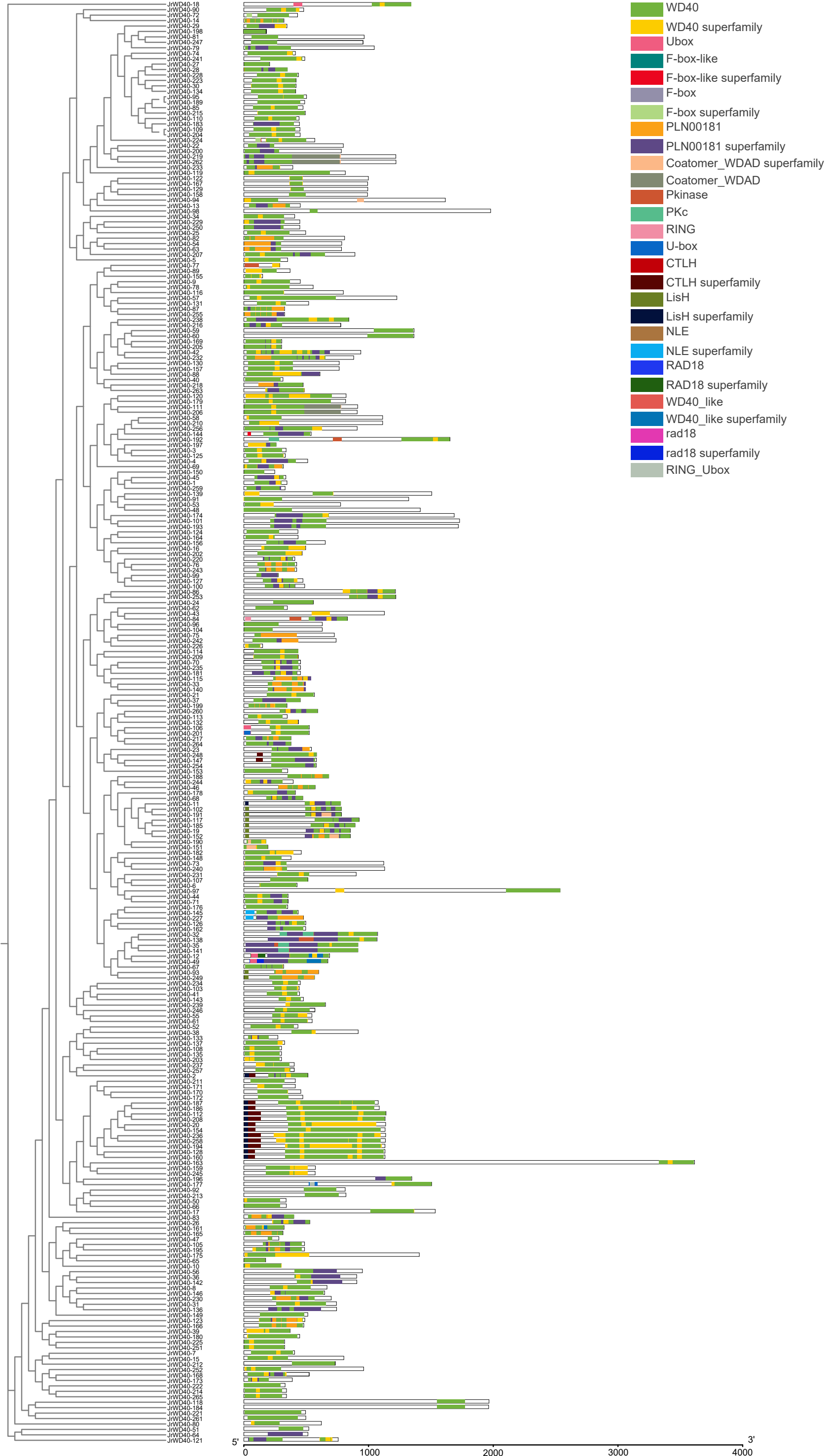

Supplement: Supplementary file 1 [file ijms-26-01071-s001.zip › Figure S2.pdf]

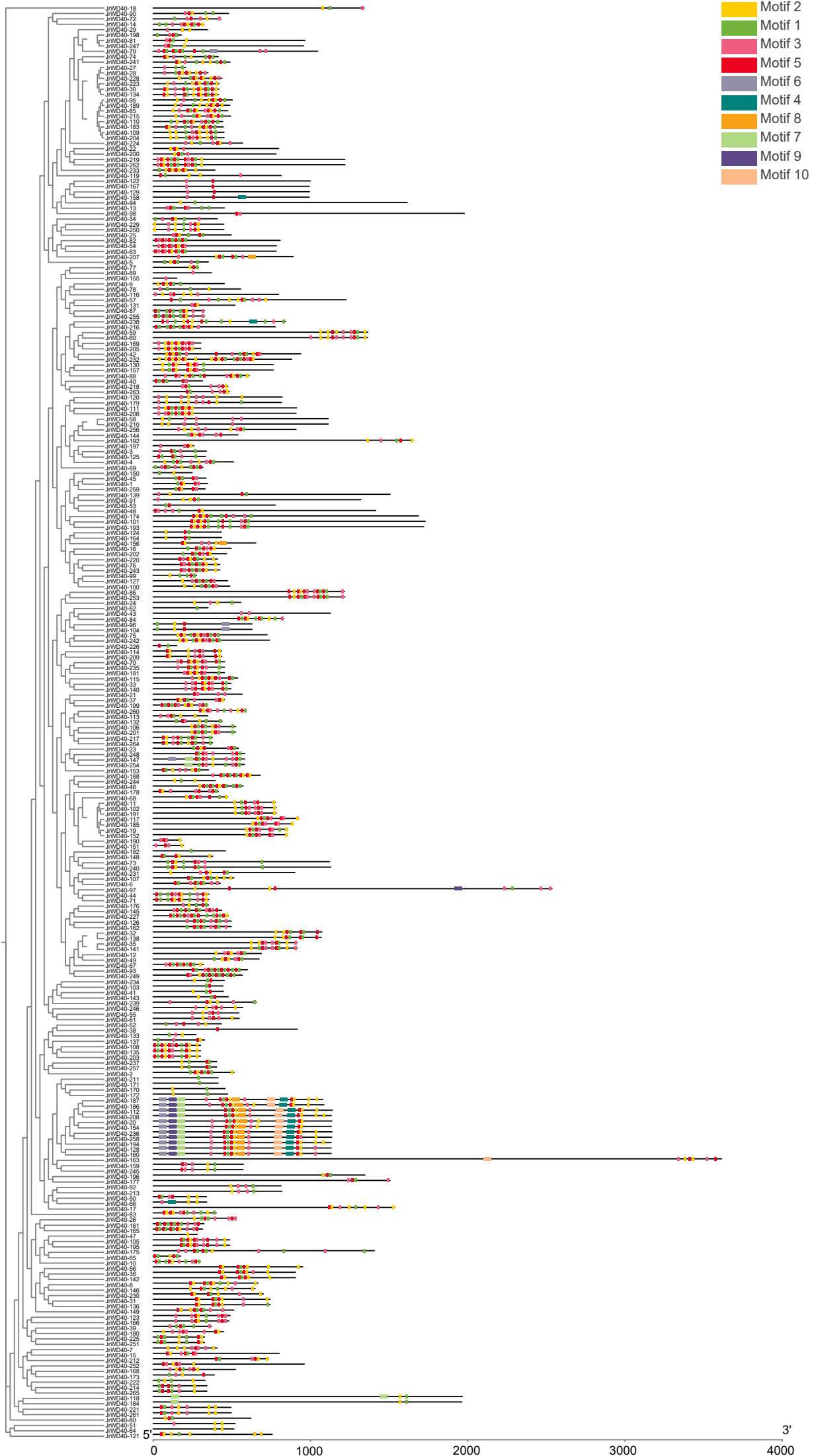

Supplement: Supplementary file 1 [file ijms-26-01071-s001.zip › Figure S3.pdf]

motif 1

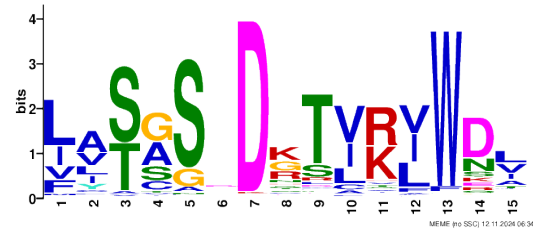

motif 2

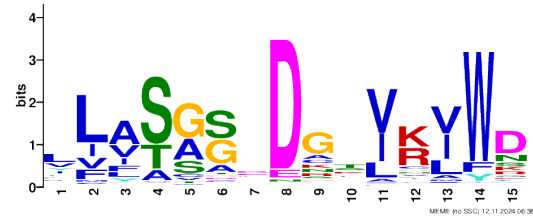

motif 3

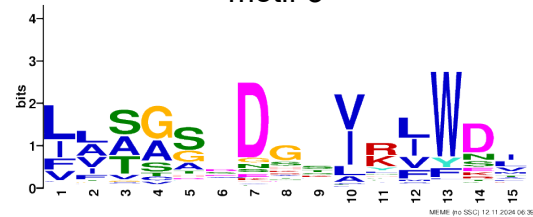

motif 4

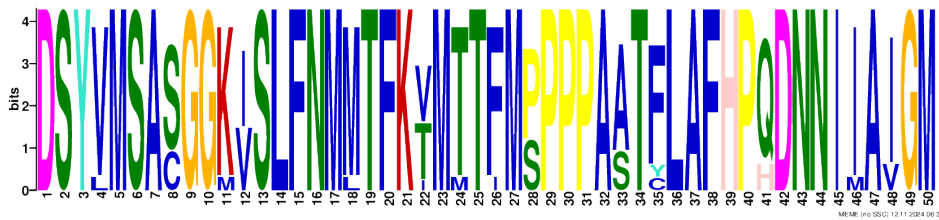

motif 5

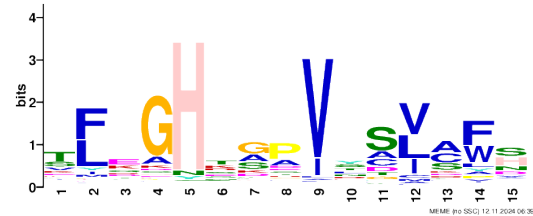

motif 6

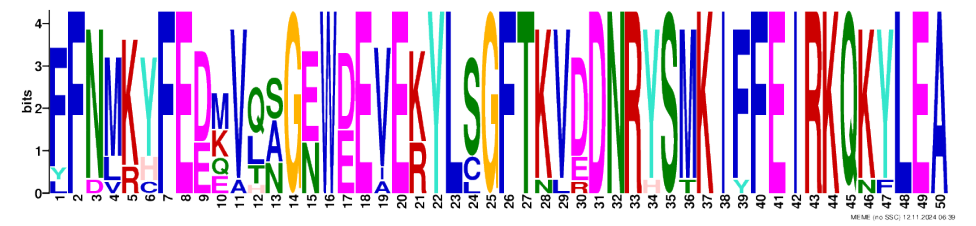

motif 7

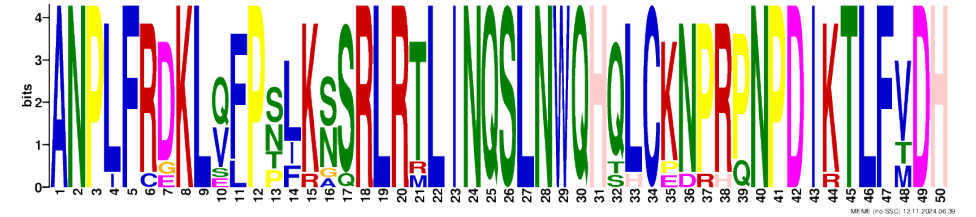

motif 8

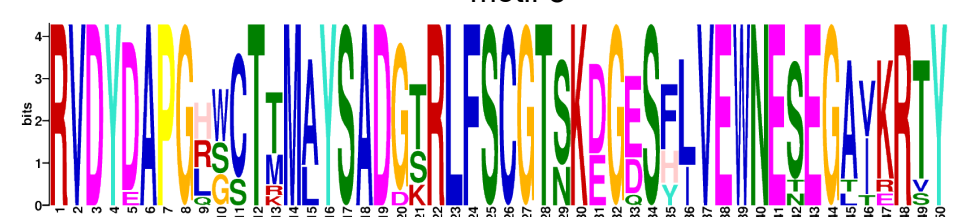

motif 9

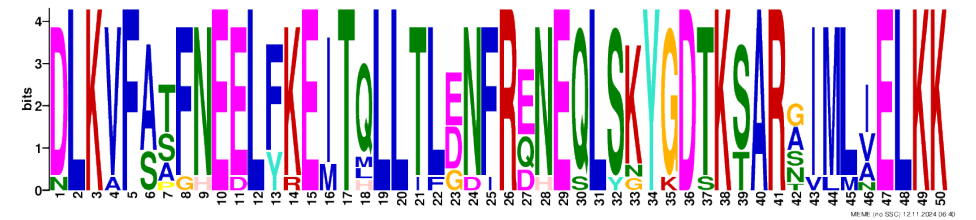

motif 10

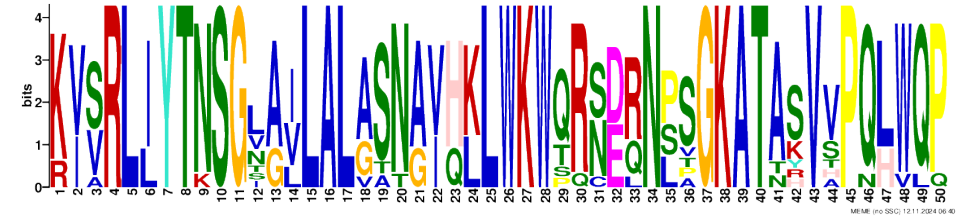

Supplement: Supplementary file 1 [file ijms-26-01071-s001.zip › Figure S4.pdf]

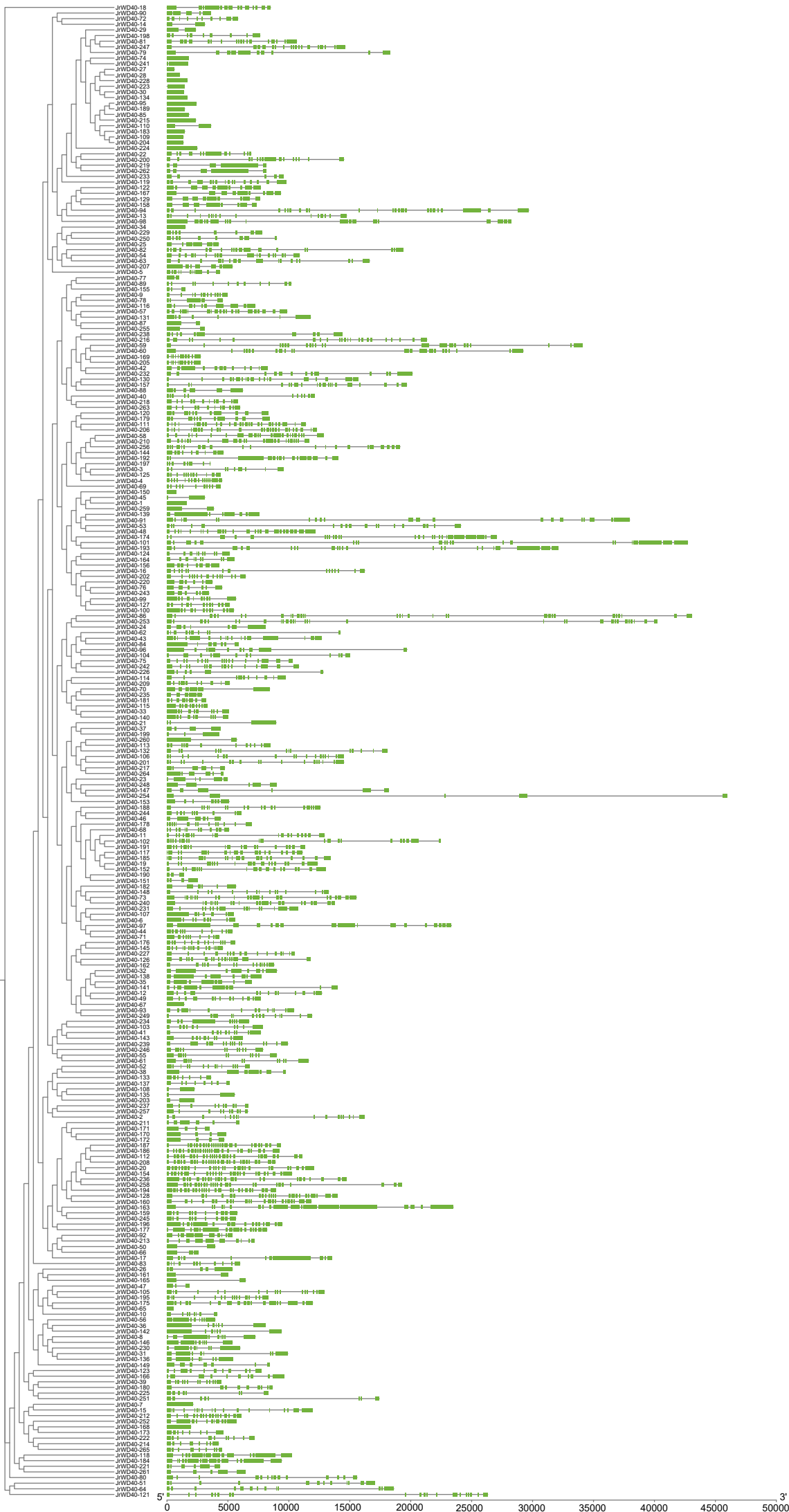

Supplement: Supplementary file 1 [file ijms-26-01071-s001.zip › Figure S5.pdf]

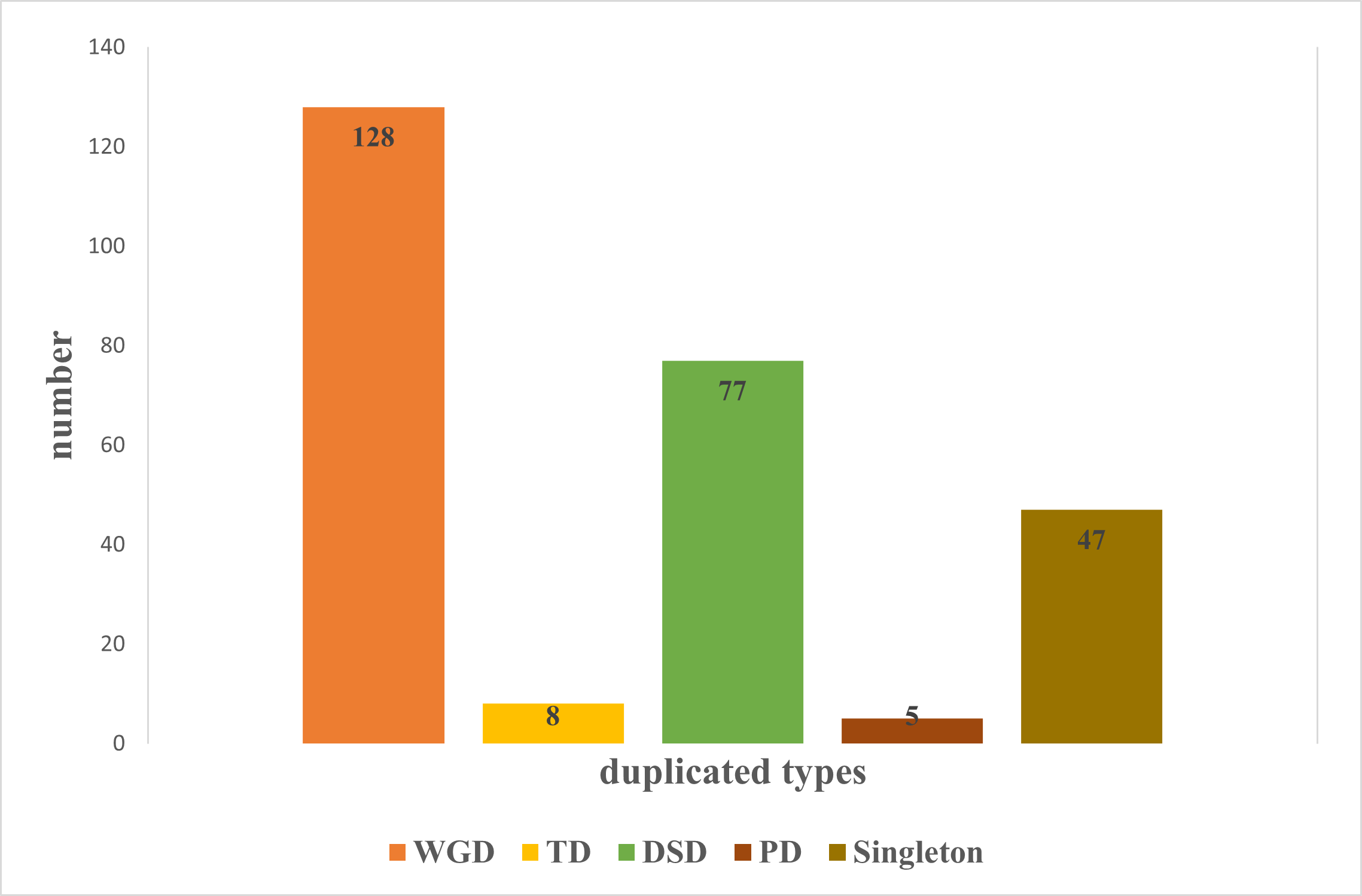

Supplement: Supplementary file 1 [file ijms-26-01071-s001.zip › Figure S6.tif]
